# Optimization of emergency allocation of necessities of life based on fractal perspective

**DOI:** 10.3389/fpubh.2023.1245415

**Published:** 2023-09-12

**Authors:** Hong Li, Zhe Zhou, Chuan Hu, Yumei Ning, Zihao Wang, Hua Huang, Kangsheng Tao

**Affiliations:** ^1^Department of Economics and Social Development, Gansu Normal College for Nationalities, Hezuo, Gansu, China; ^2^School of Business Administration, Zhongnan University of Economics and Law, Wuhan, Hubei, China; ^3^School of Public Health and Management, Wenzhou Medical University, Wenzhou, Zhejiang, China; ^4^Business School of Hbue, Hubei University of Economics, Wuhan, Hubei, China

**Keywords:** necessities of life, emergency allocation, optimization model, demand disturbance, fractal perspective

## Abstract

The supply and reserves of emergency necessities of life are important for emergency management in disaster events. The scope of the necessities of life changes with social development, and their reserves and allocation in sudden disaster events continually face new challenges. Timely distribution of the necessities of life during disasters is critical to saving lives and maintaining social order. Therefore, this study proposes a fractal multi-level distribution network (FMDN) optimization model with multiple warehouse points, multiple emergency distribution centers, and multiple disaster points from the perspective of fractal theory. The FMDN model considers the influence of road damage on vehicle speed and the dynamic change in demand at the affected points. The FMDN model aims to minimize the operating costs of a distribution network, including the cost of building emergency reserve points, transportation costs, and penalty costs for lack of demand. Numerical experiments verify the feasibility of the model. The FMDN model is solved using LINGO software programming, and an optimal distribution path and quantity are obtained. Analyzing the numerical example results shows that the model is suitable for emergencies and has good applicability.

## 1. Introduction

Since the twenty-first century, the frequent occurrence of large-scale sudden disasters has caused huge economic costs to global society. Notably, China is one of the countries most affected by frequent disasters. The United Nations *Global Assessment Report on Disaster Risk Reduction* points out that global disaster risk events are on the rise, especially natural disaster losses caused by climate change and environmental damage, causing the world $2.5 trillion in direct losses this century. Earthquakes and tropical storms cause direct losses of more than 180 billion US dollars a year globally. According to the *Economists War-Game Pandemic Threat to Global Growth* analysis report, the outbreak of the public health emergency of COVID-19, which created a pandemic, may ultimately destroy 1% of global GDP and cost global GDP more than $1.1 trillion (1).

In recent years, the number of disasters worldwide has been increasing. For example, on the morning of 7 February 2021, a glacier in the Jamori district of the Indian state of Uttarakhand collapsed, triggering massive flooding that left more than 207 people dead or missing and forcing the emergency evacuation of thousands of people from surrounding areas. In June–July 2021, the United States and Canada experienced historic heat and drought. The heat and drought resulted in more than 1,319 deaths in the U.S. and Canada, thousands of people seeking emergency medical treatment for heat-related discomfort and illness, and more than 58 million people affected by the heat and drought. On 16 December 2021, Typhoon Rey struck the Philippines, killing 378 people, leaving 60 missing and 742 injured, and affecting 3.95 million people.

Major natural disasters in China in recent years include the following: On 12 May 2008, a massive earthquake measuring 8.0 on the Richter scale struck Wenchuan County, Sichuan Province, killing 69,227 people, injuring 374,643, and leaving 17,923 missing. On 14 April 2010, an earthquake measuring 7.1 on the Richter scale struck Yushu, Qinghai, killing 2,698 people and leaving 270 missing. On 7 August 2010, a mudslide in Zhouqu County, Gannan Prefecture, killed 1,463 people, left 302 missing, and injured 72. On 3 August 2014, an earthquake measuring 6.5 on the Richter scale struck Ludian County, Yunnan Province, affecting 1,088,400 people and killing 617. With the development of urbanization, China’s main population distribution is one of the spatial aggregations characterized by urban agglomerations and super-large urban agglomerations. During large-scale emergencies, the demand for rescue materials increases geometrically in the short term. The demand structures for medical materials, necessities, emergency rescue materials, and restoration and reconstruction materials in disaster areas are complex, and the types and quantities of material demand at different stages show large fluctuations and dynamic uncertainties. This makes it difficult for emergency management to accurately predict material needs at affected sites and often leads to bottleneck problems, such as configuration delays.

The main research content of fractal theory is the scale distribution characteristics of fractal systems, that is, using mathematical methods to reveal the intrinsic scale invariance of irregular shapes in nature and to analyze the original systems’ characteristics and intrinsic evolution laws. The application of fractal theory in the distribution of necessities of life helps the regional fractal distribution units integrate and utilize limited resources, make independent decisions, adapt and optimize themselves, and complete the superior distribution tasks on time.

This study focuses on life necessities’ connotation, category, demand, and distribution characteristics. Considering the uncertainty of demand quantity, the fractal theory was adopted to efficiently transport various life necessities from warehouses to the disaster area to minimize the total delivery time. It is vital to meet the basic needs of people in disaster-stricken areas concerning the necessities of life and to maintain social order there.

## 2. Literature review

### 2.1. Literature review on necessities of life

The necessities of life are defined in both a broad and a narrow sense. In the broad sense, they refer to goods that meet people’s basic living needs, and the broad sense is increasing in scope: In a developing social economy, with advances in social production technology and an increase in product use, non-life necessities are gradually transformed into life necessities over a long period of time. In the narrow sense, the necessities of life comprise four categories: clothing, food (including firewood, rice, oil, salt, and sanitation), housing (temporary rental housing available for replacement), and transportation (e.g., public transportation). These constitute the basic means of subsistence, the basic requirements for human beings to live their lives.

Pantazis et al. (2–5) found that British people’s ideas on what life necessities comprised were usually broader and more multidimensional than those assessed by experts. Their social activities, roles, and relationships were key factors that determined the content of their necessities of life. Gordon et al. (6) believed that public identification with a range of social activities, roles, and relationships was an important factor in determining the necessities of Guernsey residents. Necessities perceived by the public depend on the interaction between the market availability and the social development structure on which the current living environment depends. Fahmy et al. (7) argued that the extent to which there is a clear public consensus on the necessities of life needs to be further explored and suggested that additional methods be applied to determine public perceptions.

Research on the necessities of life at home and abroad focuses on the establishment and improvement of emergency reserve systems, the improvement of relevant emergency planning systems, and the integrated optimization modeling of the location-routing problem (LRP) of emergency distribution centers. Teimoury et al. (8) studied the vegetable import quota problem in Tehran city through system dynamics model simulation. Brady (9) simulated the coordination and response of information flow and medical, police, and fire resources to help emergency managers quickly develop robust emergency plans to deal with potential threats.

### 2.2. Literature review on fractal theory

The fractal theory has been applied to image processing, universe exploration, financial analysis, medical diagnosis, earthquake research, and logistics. Guo et al. (10) and Bocewicz et al. (11) studied the scale characteristics of public transport networks (PTNs) in L-space through fractal analysis and considered the effect of the real bus routes, providing new perspectives and tools for human migration in spatial networks. Saad and Bahadori (12) proposed a new information fractal framework to study the improvement in the sustainability of an entire food distribution chain using two variables: the greenfield service limit and the minimum-on-board-vehicle-weight fill level. Webber and Dunbar (13) studied the fractal structure of the distribution of communities of practice and analyzed the implications for the business management structure. Zhang and Li (14) studied the multifractality of traffic flow at the spatial scale and used it to quantify the uniformity of the flow’s spatial distribution. Li et al. (15) combined a fractal method with a passenger flow allocation model to establish a fractal quantification method to measure the temporal and spatial distribution and the characteristics of passenger flow in rail transit networks. Man and Chen (16) studied the fractal and fractal dimension characteristics of Shenzhen, China. Shao et al. (17) studied the multifractality of three major cryptocurrencies using multifractal detrended fluctuation analysis (MFDFA).

Fractal theory for the study of finance has also been studied by many scholars (18–21). Jiang et al. (22) used multiple fractals to quantify financial market inefficiencies in the context of risk management. Fractal theory has also found application in the field of logistics distribution. Jingwen (23) studied the feasibility of using fractal theory to design the spatial organization of logistics companies. Ryu et al. (24) proposed a fractal-based inventory management (fVMI) model to minimize inventory costs and satisfy customer demand. Yue et al. (25) proposed a fractal hierarchical honeycomb structure for vehicle routing problem (VRP) solving. Bi et al. (26) constructed a correlation model of regional logistics dynamics using multifractal theory; they studied the coupling relationship between regional logistics dynamics and multifractal dynamics using empirical analysis.

A number of scholars have studied the optimization of material distributions under uncertain demand. Wu and Peng (27) proposed a chance-constrained model with uncertain customer demand and center-setting costs, constructing and solving the model using uncertainty theory. Wen et al. (28) studied the facility location-allocation (FLA) problem in a random environment, established an uncertain expected value model based on uncertain measures, and used a hybrid intelligence algorithm to solve numerical examples. Liu et al. (29) studied the problem of locating a multi-product logistics distribution center in an uncertain environment and constructed a total annual cost model to minimize construction, management, inventory, and transportation costs; they validated the model with numerical examples using Lingo software. Xu and Qi (30) established a dual-objective mixed-integer linear programming model to solve the problem of multi-point gasoline-emergency-distribution vehicle-path optimization with vehicle sharing and time window coordination. Cui et al. (31) proposed an objective function to minimize operating costs under uncertain demand and established a logistics center location optimization model with a three-node expansion mechanism. Xing et al. (32) introduced the fractal theory into the emergency organization construction of coastal cities in China. They proposed a virtual network fractal emergency organization structure with self-organization, self-optimization, and self-affinity characteristics. Using Typhoon “Lichma” as an example, they verified the efficient, dynamic, and open performance of virtual network fractal emergency organizations in adapting to changes in disaster scenarios. Li et al. (33) applied computational mathematical organization theory to construct a fractal emergency organization optimization model. Li et al. (34) studied the emergency response collaborative organization model and proposed an unconventional emergency response organization model based on fractal theory.

The current research on emergency necessities is inadequate. The public’s perception of the content of the necessities of life needs to be redefined using a new methodology. With the development of technology and social progress, the scope of necessities is expanding, and the distribution of emergency necessities in sudden disasters consequently faces new challenges. This study, therefore, applies fractal theory to an emergency distribution network model of the necessities of life to examine these challenges.

## 3. Fractal-based emergency allocation framework for life necessities

The American mathematician Benoit B Mandelbrot (35) defined fractals as follows: “Fractals are geometric figures or natural shapes composed of parts that resemble the whole in some way.” Fractals have the following characteristics: (a) Parts of a figure or object in a fractal are the same as the whole formal structure, but different in size, and may be slightly deformed. (b) Regardless of the level of examination, a fractal’s form is either extremely irregular, highly discontinuous, or fragmented. (c) A fractal contains some “special elements” that vary greatly in rank and cover an extremely wide range.

**Figure 1:**
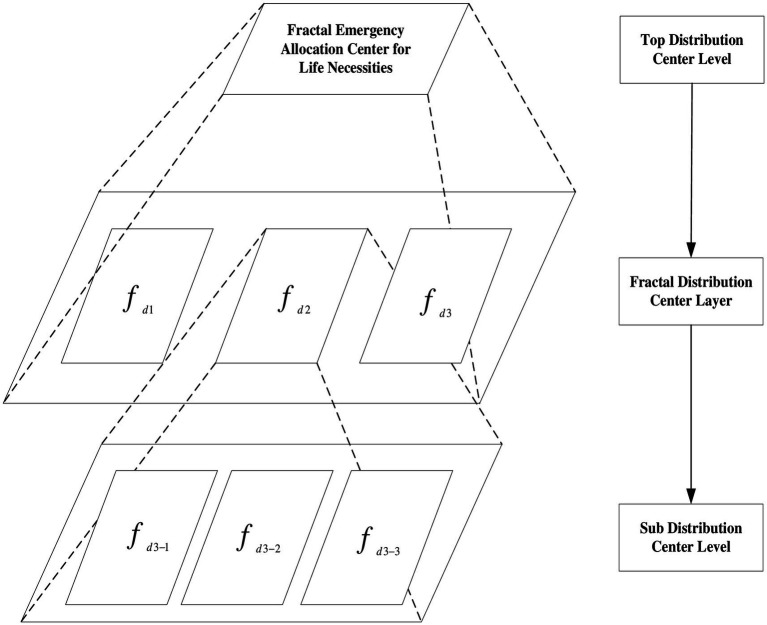
Fractal emergency allocation framework for life necessities.

Figure 1 shows the framework for establishing the emergency allocation of life necessities. A fractal distribution center framework is proposed to solve the problem of emergency configuration of life necessities; its core purpose is to obtain a function of self-organization, self-similarity, and self-optimization of sub-fractal units. Both the fractal distribution center layer and the sub-distribution center layer adopt a goal-driven mechanism: On the one hand, they are subject to the top-level distribution center goal, and on the other hand, they can optimize their internal processes and dynamically adapt to changes in the external environment and changes in the top-level goals. Fractal elements are autonomous units with autonomous decision-making, autonomous adaptation, and the execution of top-level distribution tasks.

Fractal elements are similar to the whole in some form, and a fractal emergency configuration center for necessities includes a top-level distribution center layer, a fractal distribution center layer, and a sub-distribution center layer. The top-level distribution center layer is the decision-making command center for emerging fractal configurations of the necessities of life. Generally, government agencies play this role and issue specific orders for the location and quantity of specific warehouses for the transportation of necessities during emergencies. The fractal distribution center layer is a combination of distribution platform centers that include various entities (e.g., governments, enterprises, logistics, and alliances) in a region. The fractal distribution center layer is composed of a combination of different distribution modes (e.g., third-party logistics and fourth-party logistics), whose common goal is to accomplish the task of emergency distribution of necessities. The bottom layer is the sub-distribution center layer, which is responsible for the execution of distribution tasks. The sub-distribution center layer is a combination of various fractal enterprise distribution platform centers, government fractal distribution platform centers, and fractal logistics alliance centers, providing emergency allocation of necessities of life.

Unlike previous studies, the fractal emergency distribution framework of the necessities of life can decompose the distribution tasks and alleviate the distribution pressure in a short time. Under the goal-driven mechanism, the sub-fractal units make independent decisions, adapt, and organize themselves independently, which can timely and reliably achieve the distribution goal of the top-level decomposition. The key feature is that, on the one hand, fractal distribution units increase the potential scope of collaborative units. On the other hand, the fractal distribution unit makes independent decisions and optimizes independently, which saves resources and improves distribution efficiency while achieving goals.

## 4. Optimization model for the emergency allocation of life necessities under demand disturbance

### 4.1. Problem description

**Figure 2:**
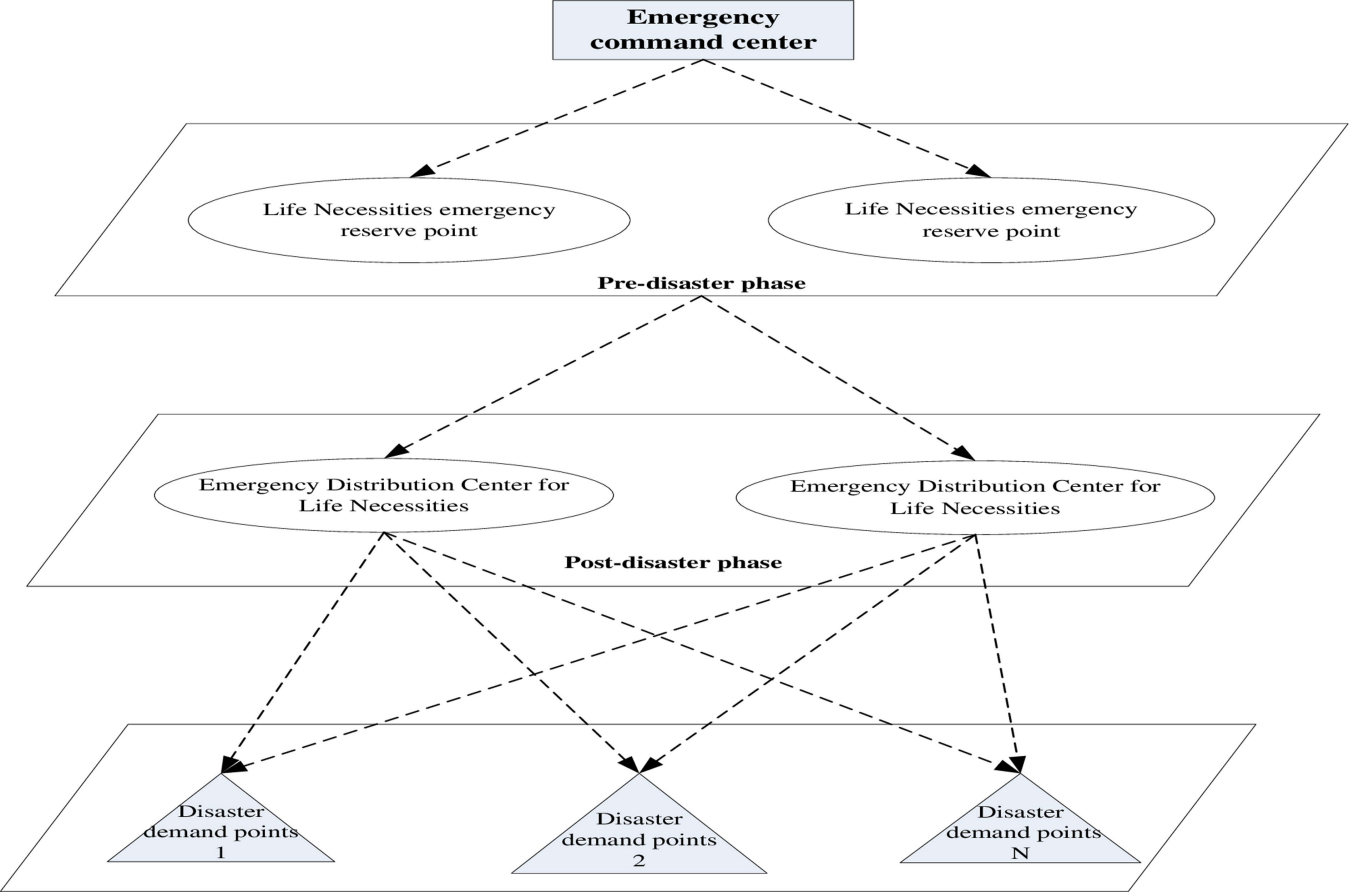
Fractal emergency distribution network for life necessities.

In large-scale emergencies, due to the limited supply of life necessities near the disaster site, it is necessary to send them from the emergency reserve point to the necessities’ distribution center as soon as possible. Emergency necessities first arrive at the emergency distribution center and are then distributed to each emergency demand point. The problem is to construct a fractal emergency distribution network optimization model that encompasses multiple emergency storage points, multiple emergency distribution centers, and multiple disaster demand points. The model must take into account the construction costs of emergency reserve points, emergency distribution center costs, transportation costs, and demand shortage penalty costs. The road condition coefficient and the penalty cost of lack of demand are introduced into the model. Finally, to minimize the operating costs of the emergency distribution network, an emergency allocation optimization model is constructed; this model is for necessities with uncertain demand and takes into account road access conditions. The fractal emergency distribution network of necessities of life is shown in Figure 2.

### 4.2. Model assumptions

(1) During the emergency rescue period, the carrying capacity of vehicles at each stage is limited, and the vehicles adopt a round-trip distribution mode.

(2) In the case of a road network interruption, there is at least one other path to reach the disaster point. The road condition coefficient influences the delivery time and speed. Basic road condition information (travel time, travel cost, and road condition coefficient) is known.

(3) Different kinds of emergency necessities are packaged and transported by independent vehicles.

(4) The end of the rescue mission is marked by the receipt of no less than the required amount of necessities at each emergency demand point.

### 4.3. Variable description

*R* is the set of alternative nodes for emergency reserve points (*r* ∈ *R*).

*D* is the set of alternative nodes for emergency distribution centers (*d* ∈ *D*).

*Q* is the set of emergency demand points for life necessities (*q* ∈ *Q*).

*M* is the set of emergency necessity categories (*m* ∈ *M*).

*K* is the set of fractal distribution stages for emergency life necessities (*k* ∈ *K*).

$C_{trm}^{k}$ is the fixed cost of establishing the *r*th emergency reserve point for Category *m* of life necessities in stage *k*.

$C_{tdm}^{k}$ is the fixed cost of establishing the *d*th emergency distribution center for Category *m* of life necessities in stage *k*.

$Y_{rdm}^{k}$ is the unit mileage transportation cost of distributing life necessities *m* from the emergency reserve point *r* to the emergency distribution center *d* in stage *k*.

$Y_{dqm}^{k}$ is the unit mileage transportation cost of distributing life necessities *m* from the emergency distribution center *d* to the emergency demand point *q* in stage *k.*

$S_{rm}^{k}\;$ is the supply of life necessities *m* from the emergency reserve point *r* in stage *k*.

$d_{qm}^{k}$ is the actual demand for life necessities *m* at the emergency demand point *q* in stage *k.*

$L_{rd}$ is the shortest distribution distance from the emergency reserve point *r* to the emergency distribution center *d.*

$\xi_{qm}^{k}$ is the penalty price per unit of shortage of emergency life necessities *m* at the emergency demand point *q* in stage *k.*

$X_{rdm}^{k}$ is the distribution volume of emergency life necessities *m* from the emergency reserve point *r* to the emergency distribution center *d* in stage *k.*

$X_{dqm}^{k}$ is the distribution volume of emergency life necessities *m* from the emergency distribution center *d* to the emergency demand point *q* in stage *k.*

$V_{dqm}^{K}$ is the delivery speed of life necessities *m* from the emergency distribution center *d* to the emergency demand point *q* in stage *k.* As the existing traffic routes are often damaged after emergencies, the normal speed of the distribution vehicles for life necessities arriving in the disaster area will inevitably be affected. In this study, the average speed of the vehicles is represented by $\bar{V_{dqm}^{k}}$ .The formula is given as follows:


(1) $$V_{dqm}^{k}=\beta_{dq}^{k}*\bar{V_{dqm}^{k}}$$

in Constraint (1), $\beta_{dq}^{k}$ is the road condition coefficient for the emergency distribution center *d* to emergency demand *q* in stage *k.* $\;\beta_{dq}^{k}\in$ [0,1]; the larger the value, the better the traffic condition of the road.

$t_{dqm}^{k}$ is the delivery time of life necessities *m* from the emergency distribution center *d* to emergency demand *q* in stage *k.*

${\hat{E}}_{qm}^{k}$ is the shortage of life necessities *m* at emergency demand *q* in stage *k.*

$f_{rm}^{k}\;\mathrm{is}$ the binary variable. When life necessities *m* are selected at the emergency reserve point *r* in stage *k*, it equals 1; if not, it equals 0.

$f_{dm}^{k}\;$ is the binary variable. When life necessities *m* are selected at the emergency distribution center *d* in stage *k*, it equals 1; if not, it equals 0.

### 4.4. Model building

The occurrence of a sudden catastrophic event can result in the destruction of transportation routes, and the speed of material distribution vehicles can be seriously affected. This model uses a road condition coefficient and demand disturbance parameters to solve for the optimum distribution route and optimum distribution quantity of an emergency distribution network.

A fractal multi-stage emergency distribution network model that considers road traffic conditions and constant demand. Hence, we have the resulting model:


(2) $$\begin{array}{c} \min Z=\underset{r\in R}{\sum }\underset{m\in M}{\sum }\underset{k\in K}{\sum }C_{trm}^{k}f_{rm}^{k}+\underset{r\in R}{\sum }\underset{d\in D}{\sum }\underset{m\in M}{\sum }\underset{k\in K}{\sum }Y_{rdm}^{k}L_{rd}X_{rdm}^{k}\\ \;+\underset{d\in D}{\sum }\underset{m\in M}{\sum }\underset{k\in K}{\sum }C_{tdm}^{k}f_{dm}^{k}\\ \;+\underset{d\in D}{\sum }\underset{q\in Q}{\sum }\underset{m\in M}{\sum }\underset{k\in K}{\sum }Y_{dqm}^{k}\beta_{dq}^{k}t_{dqm}^{k}\bar{v_{dqm}^{k}}X_{dqm}^{k}\\ \;+\underset{q\in Q}{\sum }\underset{m\in M}{\sum }\underset{k\in K}{\sum }{\hat{E}}_{qm}^{k}\xi_{qm}^{k} \end{array}$$

(3) $$\underset{d\in D}{\sum }X_{dqm}^{k}\geq d_{qm}^{k}+{\hat{E}}_{qm}^{k},q\in Q,m\in M,k\in K$$

(4) $$\underset{r\in R}{\sum }X_{rdm}^{k}\leq S_{rm}^{k}f_{rm}^{k},d\in D,\;\;m\in M,k\in K$$

(5) $$\underset{r\in R}{\sum }X_{rdm}^{k}=\underset{d\in D}{\sum }X_{dqm}^{k},q\in Q,\;\;m\in M,\;\;k\in K$$

(6) $$\underset{r\in R}{\sum }f_{rm}^{k}\geq 1,m\in M,\;\;k\in K$$

(7) $$\underset{d\in D}{\sum }f_{dm}^{k}\geq 1,m\in M,\;\;k\in K$$

(8) $$f_{rm}^{k}\in \left\{0,1\right\},r\in R,m\in M,k\in K$$

(9) $$f_{dm}^{k}\in \left\{0,1\right\}d\in D,\;\;m\in M,\;\;k\in K$$

(10) $$X_{rdm}^{k}\geq 0,r\in R,d\in D,m\in M,k\in K$$

(11) $$X_{dqm}^{k}\geq 0,d\in D,q\in Q,m\in M,k\in K$$

(12) $${\hat{E}}_{qm}^{k}\geq 0,q\in Q,\;\;m\in M,\;\;k\in K$$

Constraint (2) is the objective function. The formula aimed to minimize the total cost of the emergency distribution network by adding volume-shortage parameters and its penalty cost. The total cost includes the fixed construction cost of the emergency reserve point, the transportation cost from the emergency reserve point to the emergency distribution center, the fixed construction cost of the emergency distribution center, the transportation cost from the emergency distribution center to the emergency demand point, and the penalty cost of the demand shortage at the emergency demand point. Constraint (3) states that the number of necessities allocated to each emergency demand point should satisfy the demand as much as possible in stage *k*. Constraint (4) is that in stage *k*, the distribution quantity of the necessities of life *m* from the emergency reserve point *r* to the emergency distribution center *d* should be less than or equal to the supply quantity of the necessities of life *m* supplied by the emergency reserve point *r*, and only the selected emergency reserve point can distribute. Constraint (5) is the sum of the emergency life necessities supplied from the emergency reserve point, equal to the sum of the delivery volume of the emergency distribution center. Constraint (6) established at least one emergency reserve point for life necessities in the emergency distribution network in stage *k*. Constraint (7) established at least one emergency distribution center for life necessities in the emergency distribution network in stage *k*. Constraints (8) and (9) are decision variable constraints. Constraints (10), (11), and (12) are non-negative constraints.

In reality, large-scale emergencies lead to dynamic changes in the number of emergency life necessities demanded in disaster areas. In the objective function (2), it is difficult to accurately simulate the number of necessities of life under disaster by studying the emergency distribution network model with determined demand. Since the solution to constraint (2) of the objective function is easy to find, constraint (2) is not solved further.

To accurately simulate the changes in demand for life necessities during a disaster event, the following analysis is performed:

In this study, the following interval estimates are given for the demand for different categories of life necessities *m* in stage *k*:

$d_{qm}^{k}\in$ [ $\bar{d_{qm}^{k}}$ , $\overset{⏜}{d_{qm}^{k}}$ ] (*q* ∈ *Q*, m∈M , *k* ∈ *K*), where $\bar{d_{qm}^{k}}$ is the nominal demand value of life necessities *m* at demand point *q* in stage *k*. $\overset{⏜}{d_{qm}^{k}}$ is the maximum disturbance value that deviates from the nominal value. $\overset{⌢}{d_{qm}^{k}}/\bar{d_{qm}^{k}}$ is the disturbance coefficient.

In emergencies, the probability of the number of necessities of life reaching the limit is extremely low. That is, when $d_{qm}^{k}$ = $\bar{d_{qm}^{k}}$ , the demand forecast is insufficient. When $d_{qm}^{k}$ = $\bar{d_{qm}^{k}}$ + $\overset{⌢}{d_{qm}^{k}}$ , there is a surplus of emergency life necessities. In this study, a control parameter $\gamma_{qm}^{k}\;$ is introduced to control the disturbance of the demand for life necessities at each emergency demand point in the interval $\left[\bar{d_{qm}^{k}},\overset{⌢}{d_{qm}^{k}}\right]:$

(13) $$H_{qm}^{k}=\frac{d_{qm}^{k}-\bar{d_{qm}^{k}}}{\overset{⌢}{d_{qm}^{k}}}\leq \gamma_{qm}^{k}\;\left(q\in Q,m\in M,k\in K\right)$$

By introducing the control parameter $\gamma_{qm}^{k}$ into the model, the solution infeasibility that occurs when demand $d_{qm}^{k}$ varies within an interval is avoided. If $\gamma_{qm}^{k}$ =0, then $d_{qm}^{k}$ = $\bar{d_{qm}^{k}}$ is a deterministic problem with demand equal to the lower bound of the interval. if $\gamma_{qm}^{k}$ =1, then $d_{qm}^{k}$ = $\bar{d_{qm}^{k}}+\overset{⌢}{d_{qm}^{k}}$ is an absolutely robust correspondence problem. Therefore, the range of demand disturbance can be controlled by adjusting the value of $\gamma_{qm}^{k}$ between the interval [0,1] . This provides a model solution for demand disturbance. Thus, constraint (3) can be transformed into the following:


(14) $$\underset{d\in D}{\sum }X_{dqm}^{k}\geq \overset{⏜}{d_{qm}^{k}}+\gamma_{qm}^{k}d_{qm}^{\hat{k}}+{\hat{E}}_{qm}^{k}\left(q\in Q,m\in M,k\in K\right)$$

Therefore, a fractal multi-stage emergency allocation optimization model of life necessities is built considering road traffic conditions under demand disturbance:


(15) $$\begin{array}{c} \min Z=\underset{r\in R}{\sum }\underset{m\in M}{\sum }\underset{k\in K}{\sum }C_{trm}^{k}f_{rm}^{k}+\underset{r\in R}{\sum }\underset{d\in D}{\sum }\underset{m\in M}{\sum }\underset{k\in K}{\sum }Y_{rdm}^{k}L_{rd}X_{rdm}^{k}\\ \;\;+\underset{deD}{\sum }\underset{meM}{\sum }\underset{keK}{\sum }C_{trm}^{k}f_{rm}^{k}+\underset{d\in D}{\sum }\underset{q\in Q}{\sum }\underset{m\in M}{\sum }\underset{k\in K}{\sum }Y_{dqm}^{k}\beta_{dq}^{k}t_{dqm}^{k}\\ \;\;\;\bar{v_{dqm}^{k}}\;\left(\bar{d_{dqm}^{k}}+\gamma_{qm}^{k}\overset{⌢}{d_{qm}^{k}}+{\hat{E}}_{qm}^{k}\right)\\ \;+\underset{q\in Q}{\sum }\underset{m\in M}{\sum }\underset{k\in K}{\sum }{\hat{E}}_{qm}^{k}\xi_{qm}^{k} \end{array}$$

Subject to Constraint (4) ~ Constraint (14).

## 5. Numerical experiments

**Figure 3:**
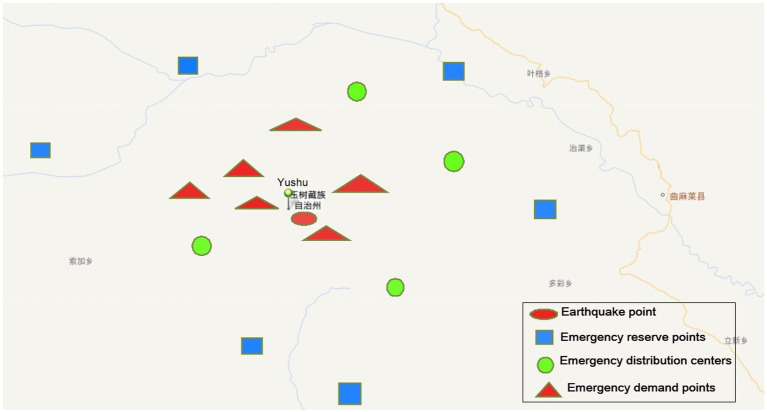
Regional disaster map.

In the 7.1 Richter scale earthquake that struck Yushu City, Qinghai Province, China, the emergency distribution of necessities, such as food, medicine, and tents, was urgently needed by the victims. Let there are six life necessities reserve points, four regional emergency distribution centers, and six emergency demand points in the provincial, prefectural, and municipal emergency distribution network that require the supply of life necessities, as shown in Figure 3. The minimum number of emergency distribution centers in the emergency allocation network is 2 (Tables 1–9). The relevant data are as follows:

**Table 1:** Fixed cost of emergency reserve points (unit: 10,000 CNY) and quantity of life necessities supplied at emergency reserve points (unit: tons).

R	r1	r2	r3	r4	r5	r6
( $C_{trm}$ )	1,000	600	3,000	1,000	2000	800
( $S_{rm}$ )	3,000	2000	5,000	3,000	4,000	1,500

**Table 2:** Fixed cost of the emergency distribution center (unit: 10,000 CNY).

( d )	d1	d2	d3	d4
( $C_{tdm}$ )	7,000	8,000	6,000	6,000

**Table 3:** Distribution distance from the emergency reserve point to the emergency distribution center (km).

D R	d1	d2	d3	d4
r1	30	23	25	26
r2	28	21	13	9
r3	17	14	15	16
r4	13	34	11	27
r5	24	12	18	33
r6	50	46	71	68

**Table 4:** Relevant values of emergency demand points.

( q )	q1	q2	q3	q4	q5	q6
$d_{qm}$	200	280	300	320	300	350
${\hat{Ε}}_{qm}$	80	90	60	30	22	27
$\xi_{qm}$	0.0025	0.0021	0.0022	0.0029	0.0026	0.0023

**Table 5:** Transportation costs from the emergency reserve point to the emergency distribution center (10,000 CNY/per ton/km).

D R	d1	d2	d3	d4
r1	6	3	6	2
r2	2	5	8	9
r3	7	4	5	6
r4	3	4	1	7
r5	4	2	8	3
r6	3	8	2	9

**Table 6:** Transportation costs from the emergency distribution center to the emergency demand point (10,000 CNY/per ton/km).

Q D	q1	q2	q3	q4	q5	q6
d1	0.09	0.058	0.08	1.10	0.079	0.04
d2	0.08	1.18	0.02	0.097	1	0.087
d3	1.12	0.056	1.14	0.089	0.05	0.07
d4	0.085	0.09	0.079	1.08	0.03	0.06

**Table 7:** Road condition coefficient from the emergency distribution center to the emergency demand point.

Q D	q1	q2	q3	q4	q5	q6
d1	0.56	0.80	0.60	0.45	0.70	0.90
d2	0.60	0.37	0.96	0.52	0.48	0.62
d3	0.42	0.81	0.39	0.63	0.85	0.78
d4	0.65	0.56	0.70	0.46	0.91	0.82

**Table 8:** Travel time of vehicles from the emergency distribution center to the emergency demand point (h).

Q D	q1	q2	q3	q4	q5	q6
d1	0.5	0.7	0.6	0.4	0.7	0.8
d2	0.6	0.3	0.9	0.5	0.4	0.6
d3	0.4	0.9	0.3	0.6	0.7	0.7
d4	0.6	0.5	0.7	0.4	0.8	0.6

**Table 9:** Average speed of vehicles from the emergency distribution center to the emergency demand point (km/h).

Q D	q1	q2	q3	q4	q5	q6
d1	25	70	54	36	61	79
d2	52	18	88	44	38	56
d3	30	72	28	58	76	68
d4	59	48	61	37	82	74

LINGO is a software developed by LINDO that can solve large-scale linear and non-linear programming problems and deal with optimization problems. In this example, to verify the model’s effectiveness, the penalty cost of the lack of quantity at the demand point in an emergency is considered, as is road damage. The model was verified and solved using LINGO software to minimize the operating cost of a three-level fractal emergency distribution network for a given class of life necessities. We used LINGO11.0 software, 8 G memory, and a 1.80 GHz AMD processor to solve the model. The model obtained optimal transportation volumes, as shown in Tables 10, 11.

**Table 10:** Model results: volume of the emergency reserve point to the emergency distribution center (unit: tons).

D R	d1	d2	d3	d4
r1	0	0	0	0
r2	0	0	0	0
r3	0	0	0	0
r4	0	0	2059	0
r5	0	0	0	0
r6	0	0	0	0

**Table 11:** Model results: transportation volume from the emergency distribution center to the emergency demand point (unit: tons).

Q D	q1	q2	q3	q4	q5	q6
d1	0	0	0	0	0	0
d2	0	0	0	0	0	0
d3	280	370	360	350	322	377
d4	0	0	0	0	0	0

The emergency reserve point obtained from the model solution is r4 , and emergency distribution centers were selected as d4 and d3 . Since the goal of the solution is to achieve the lowest total cost of the system, only one distribution center is used in the actual configuration. The total operating cost of the distribution network was 367.31 million yuan. The distribution path is (r4,d3) . Emergency life necessities are first distributed from the r4 emergency reserve point to the d3 emergency distribution center and then distributed to six emergency demand points through the d3 emergency distribution center. The specific distribution routes are shown in Table 12.

**Table 12:** Model results: emergency distribution routes.

Distribution nodes	Distribution routes					
	d1	d2	d3	d4		
R 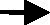 D	–	–	r4 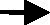 d3	–		
D 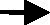 Q	q1	q2	q3	q4	q5	q6
	d3 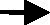 q1	d3 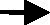 q2	d3 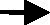 q3	d3 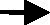 q4	d3 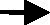 q5	d3 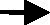 q6

To better verify the model’s performance, given a value of 0.5 for the control parameter, measure the cost and location of the emergency distribution network for necessities with a 5, 10, and 30% disturbance coefficient. Comparing the target optimal values of the stochastic demand model, the relative robust model, and the absolute robust model, the model results are shown in Table 13.

**Table 13:** Model results.

Disturbance coefficient	Absolute robust model		Stochastic demand model		Relative robust model	
	Cost	Location	Cost	Location	Cost	Location
5%	37987.28	( r4 , d3 )	37417.41	( r4 , d3 )	37359.14	( r4 , d3 )
10%	39243.55	( r4 , d3 )	38603.81	( r4 , d3 )	37987.28	( r4 , d3 )
30%	44268.65	( r4 , d3 )	41649.43	( r4 , d3 )	40499.83	( r4 , d3 )

**Figure 4:**
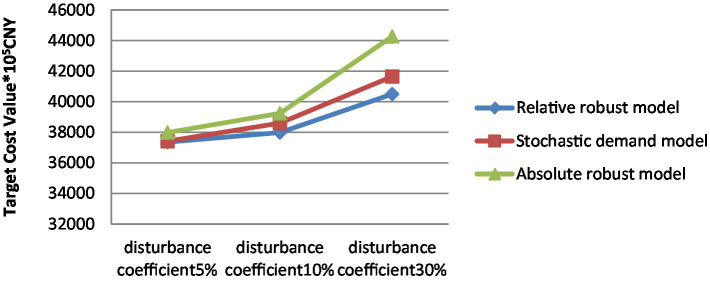
Total target cost comparison.

Figure 4 compares the total target cost values with 5, 10, and 30% disturbance coefficients. As can be seen from the comparison of the total target cost values of the model in Figure 4, as the value of the disturbance coefficient increases, the total target cost value increases, and in which the absolute robust model objective value is maximized. The deviation range of objective function values between the relatively robust and stochastic demand models is less than 5%. Therefore, it is shown that the relatively robust model achieves the optimality of the target value under uncertain demand. The robustness goal of minimum cost of the emergency distribution network under demand disturbances is achieved.

## 6. Conclusion

In the world, there is a high demand for necessities of life, with a wide variety of categories. With the development in social productivity and progress in science and technology, the scope of the necessities of life in the narrow sense is expanding. This study applies fractal theory to an emergency distribution network analysis framework and constructs a fractal-multi-stage-distribution network (FMDN) model under demand disturbance. The FMDN model includes multiple emergency reserve points, multiple emergency distribution centers, and multiple disaster points. Then, considering the actual situation of the emergency, the road condition coefficient and demand disturbance parameters are added to the FMDN model. The goal is to minimize the delivery cost of the FMDN model and solve it using LINGO software programming. Finally, a numerical experiment was solved and analyzed, and an optimal distribution path and a distribution volume were obtained. The feasibility of the model was verified.

It is important for the national and local emergency management departments to ensure the basic living and production needs of people in disaster areas are met. It is also important that the smooth implementation of emergency relief work through an efficient emergency distribution network be obtained to allocate the necessities of life to emergency demand points during large-scale emergencies. Whether life necessities can be allocated to disaster areas in time directly impacts the lives and property of those affected as well as the stability of the disaster area. We suggest that the central and local governments establish emergency reserve points based on the types of necessities of life. The government should consider the introduction of a minimum-quantity reserved system for emergency distribution centers, as well as formulating effective and feasible emergency distribution plans for essential commodities. Consideration should also be given to strengthening the existing road infrastructure of emergency distribution networks; establishing emergency linkage mechanisms across provinces, autonomous regions, and local departments to achieve inter-regional emergency linkage and collaboration; and improving the layout of emergency distribution multimodal transportation facilities to ensure the timeliness and stability of emergency distribution of life necessities in large-scale emergencies. This study provides a reference for government departments to deal with the distribution of life necessities during emergencies.

Only the allocation of emergency necessities with uncertain demand and road traffic conditions was considered in our model. We did not, therefore, take into account the perishability of life necessities, cold chain transportation, and other related factors. This should be considered in future research.

## Data availability statement

The original contributions presented in the study are included in the article/supplementary material, further inquiries can be directed to the corresponding author.

## Author contributions

CH: supervision. HL: conceptualization, formal analysis, methodology, writing the original draft, and writing, reviewing, and editing. ZZ: reviewing and editing. ZW and YN: data curation. HH and ZZ: validation. KT: visualization. HL and CH: funding acquisition. All authors contributed to the manuscript and approved the submitted version.

## Funding

This study was supported by the Phased Research Results of the Gansu Provincial Philosophy and Social Science Planning Project (2022YB122). The study was supported by the Gansu Provincial Department of Education Innovation Fund Project (2023A-150) and Research Platform for the Knowledge Mechanism of China’s Key Core Technological Innovation Ability Improvement on the Background of Dual Circulation (31513110812/108).

## Conflict of interest

The authors declare that the research was conducted in the absence of any commercial or financial relationships that could be construed as a potential conflict of interest.

## Publisher’s note

All claims expressed in this article are solely those of the authors and do not necessarily represent those of their affiliated organizations, or those of the publisher, the editors and the reviewers. Any product that may be evaluated in this article, or claim that may be made by its manufacturer, is not guaranteed or endorsed by the publisher.
